# Dietary phytate lowers *K*-*ras* mutational frequency, decreases DNA-adduct and hydroxyl radical formation in azoxymethane-induced colon cancer

**DOI:** 10.22038/IJBMS.2019.34374.8161

**Published:** 2020-01

**Authors:** Poorna Venkata Satya Prasad Pallem, Sreedhar Bodiga, Vijaya Lakshmi Bodiga

**Affiliations:** 1 Department of Biotechnology, Krishna University, Machilipatnam, Andhra Pradesh, India; 2 Department of Biochemistry, Kakatiya University, Warangal, Telangana, India; 3 Institute of Genetics and Hospital for Genetic Diseases, Begumpet, Osmania University, Hyderabad, Telangana, India

**Keywords:** Azoxymethane, Colon, DNA adducts, Hydroxyl radical, Inositol hexaphosphate K-ras

## Abstract

**Objective(s):**

Dietary phytate is known to protect against azoxymethane (AOM)-induced preneoplastic lesions. The present study was designed to determine whether dietary phytate affects mutation frequency in colon epithelial cells challenged with azoxymethane *in vivo*, through lowering the formation of O^6^-methyl guanosine (O^6^-MeG) and 8-hydroxy deoxyguanosine (8-OHdG) adducts.

**Materials and Methods:**

We used Fisher F344 rats induced with AOM for 20 weeks and undertook 1% or 2% phytate supplementation for subsequent 16 weeks to monitor the mutation frequencies of one of the candidate genes, K-*ras*, along with DNA adduct load.

**Results:**

Dietary phytate significantly suppressed aberrant crypt foci formation and effectively inhibited colon tumor formation in a dose-dependent manner. DNA sequencing results demonstrated that 60% of the colon tumors from AOM-treated and control diet fed animals showed GGT to GAT transition and 40% of the tumors showed GGT to GTT transversion at codon 12, along with 18% of the tumors showing GGC to CGC transversion at codon 13. Phytate supplementation at 1 and 2% lowered the frequency of GGT > GAT to 30 and 10%, respectively. Phytate supplementation also nullified the codon 13 mutations. No mutations were observed at codon 61 in any of the experimental groups.

**Conclusion:**

The lowered frequency of K-*ras* mutations correlated with decreased formation of hydroxyl radicals, O^5^-meG and 8-OH-dG levels in phytate-supplemented animals with lowered tumor burden.

## Introduction

Dietary phytate (Inositol hexaphosphate, IP_6_) has been investigated as a potential inhibitor of colon carcinogenesis (1, 2). Studies on dietary fibre intake and decreased incidence of colon cancer in humans have led to testing of dietary phytate as a chemopreventive agent in laboratory animal studies (2-5). Another rationale behind considering phytate is the hypothesis that iron contributes to increased proliferation and its chelation tends to suppress the tumor growth (6-8). Indeed, colon tumors were shown to accumulate iron (9), and iron deficiency was shown to decrease tumor burden (10). 

Other potential mechanism of phytate’s chemopreventive efficacy is through attenuating the mutation frequency resulting in decreased tumor burden. One such target for monitoring intervention efficacy is the *ras* oncogene. *K-ras* (Kirsten rat sarcoma oncogene homolog in rodent) mutations are observed with a frequency of 50% in human colon tumors (11), favoring the causative role of this gene in human malignancies. Increased DNA alkyl adduct formation in the colon appears to play a role in both animal and human carcinogenesis studies (12-15). The promutagenic O^6^-methyl-2-deoxyguanosine (O^6^-MeG) lesions, if left unrepaired or removed by apoptosis, results in reading of G as A by the DNA polymerase (16, 17). 8-hydroxy-2-deoxyguanosine (8OHdG), if not removed from the cell, results in G to T transversions (18). Aberrant crypt foci (ACF) are considered the early markers during colon adenoma-carcinoma transition, with accumulating *K-ras* mutations as molecular signatures (17, 19, 20). Azoxymethane (AOM)-administered rat model is rigorously employed for studying the mechanisms of colon carcinogenesis. Due to close match in terms of morphological similarity to the human tumors, despite infrequent metastases (21), this model is highly used for intervention studies.

AOM upon hydroxylation yields methylazoxymethanol (MAM), which upon alkylation results in the formation of methyldiazonium ion or methyldiazohydroxide. Relative stability of MAM (t_1/2_~12 hr) enables it to be carried to extrahepatic organs in the blood (22, 23). While AOM-induced mutations mostly result from direct DNA-carcinogen interactions, the mutagenic action of iron is believed to be indirect, namely, free radical-induced oxidative modification of DNA (24). Guanine residues in DNA can be hydroxylated to form 8-OHdG by several reducing agents or transition metals, and hydroxyl radical is implicated in this process (25). Elevated levels of 8-OHdG in tissues have been reported after treatment with reactive oxygen species (ROS)-producing carcinogen, and Fe-nitrilotriacetate (NTA) (26), which were associated with G- to T transversions *in vitro* (18, 27). Point mutations in codons 12, 13, and 61 of *K-ras* yield constitutively active KRAS protein, due to loss of regulation by the inhibitory guanosine triphosphate hydrolase activating proteins (GAPs). Lack of regulation leads to chronic activation of downstream signaling mediators, including the Raf-MEK-ERK cascade, with the end result being promotion of cellular proliferation and transformation (28, 29).

 This study was therefore undertaken to critically examine if dietary phytate can alter the mutation frequency of the *K-ras* oncogene in AOM-administered animals, while monitoring the incidence of ACF, colon adenoma/adenocarcinoma along with O^6^-MeG and 8-OHdG adducts and ROS formation.

## Materials and Methods

***Chemicals and reagents***

AOM, sodium salt of inositol hexaphosphate, O^6^MeG, methanol, acetonitrile, acetic acid, formic acid, ammonium formate, adenine, guanine and 10% (v/v) neutral buffered formalin were obtained from Sigma (St. Louis, MO, USA). All other chemicals and reagents used were of analytical grade and purchased from local sources.

***Diet and animals***

**Figure 1:**
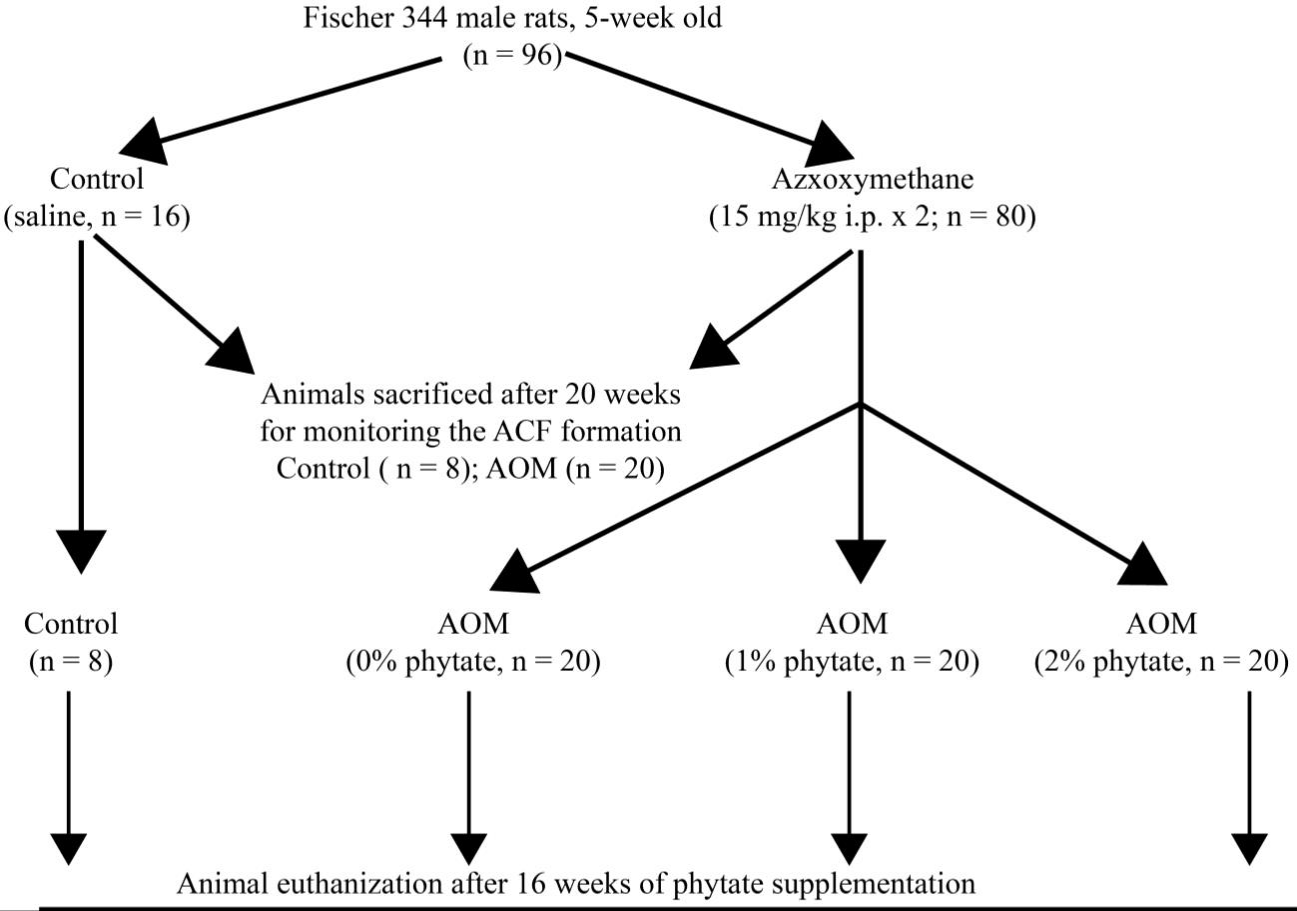
Scheme for the experimental course of the colon carcinogenesis model for the dietary administration of phytate. Carcinogenesis was induced by administration of azoxymethane (AOM). Dietary phytate was administered at 1% or 2%, at the expense of cornstarch after confirming the development of aberrant crypt foci. Number of animals in each group is indicated by ‘n’

This study was in accordance with the Institutional Animal Care and Ethics Committee (IACEC) of the National Institute of Nutrition, Hyderabad, India. A total of 96 five-week-old male Fisher 344 rats, weighing 90–100 grams were housed individually in plastic cages and in a well-ventilated room with 25-27 ^°^C, 50±10% relative humidity, and a 12-hour light/dark cycle. These animals were fed according to the American Institute of Nutrition (AIN-93G) diet recommendations *ad libitum*. Experimental design and the dietary intervention is schematically represented in Figure 1. The rats were randomly assigned to control (n=16) or AOM (n=80) groups. AOM group of rats received azoxymethane (15 mg/kg body weight; IP) diluted in physiological saline, once weekly for two consecutive weeks (30). Control rats were administered an equal volume of saline and served as the vehicle control. After 20 weeks of initiation, 8 animals from control group and 20 animals from AOM group were sacrificed to study the formation of ACF in colon. The remaining animals (n=60) in AOM group were randomly divided into 3 groups and fed either 0, 1 or 2% phytate containing AIN-93G diet for the next 16 weeks (n=20, each). Phytate was added at the expense of corn starch. The effect of dietary phytate on suppression of colonic ACF and their progression into colonic tumors was studied after 16 weeks of phytate consumption. 

***ACF analysis and tumor assessment***

At the end of study duration, ACF and tumor analysis was performed according to Bird (30). Percentage tumor incidence in each group of animals is reported, along with tumor multiplicity in tumor-bearing rats. 

***Apoptosis assay***

Terminal deoxynucleotidyl transferase (TdT) dUTP nick-end labeling (TUNEL) assay was used to detect apoptotic cells (ApopTag Peroxidase *In Situ* Apoptosis Detection Kit, Chemicon International). TdT labels the blunt ends of fragmented DNA, without the need of a template strand. Tissue sections (4-5 μm) were stained using a colorimetric system. Apoptotic index was calculated as the percentage of cells with brown labeled nuclei as against the non-apoptotic cells, which were stained blue (31). Five different sections were considered for obtaining the percentage of apoptotic cells (mean±standard error of the mean (SEM)).

***DNA extraction from paraffin-embedded tissues for monitoring K-ras mutations***

DNA was extracted from these normal or ACF tumor tissue sections using Qiagen DNeasy Tissue Kit (Qiagen, Valencia, CA). DNA was amplified by semi-nested polymerase chain reaction using primer sets designed for *K-ras* (exons 1 and 2). Negative controls without DNA were included in all reactions. PCR products were resolved on acrylamide gel and further purified using a QIAquick Gel Extraction Kit (Qiagen, Valencia, CA). These purified PCR products were amplified using Terminal Ready Reaction Mix-Big Dye (Perkin Elmer, Foster City, CA), and the amplified products were purified using DyeEx 2.0 Spin Kit (Qiagen, Valencia, CA). The PCR products were subjected to sequencing using an automatic sequencer (Perkin-Elmer ABI Model 3100). The sequence data were utilized for identifying and confirming the base changes and mutations. Primers used for amplifying the hot spot regions of rat *K-ras *gene are shown below:

**Exon           Codon           Primer                                 Strand Sequence**

 1           K-12-13           K-*ras*F25927                                 Sense 5’-ACTTGTGGTAGTTGGAGC-3’ 

                                      K-*ras*R26069                                 Antisense 5’-CTGCCACCCTTTACAAATTG-3’ 

                                       K-*ras*R26034                                 Antisense 5’-GCAGCATTTACCTCTATCGT-3’ 

 2           K-61                  K-*ras*F14325                                 Sense 5’-ATCCAGACTGTGTTTCTACC-3’ 

                                       K-*ras*R14035                                 Antisense 5’-TGCAGGCCTAACAACTAGC-3’ 

                                           K-*ras*R13986                                 Antisense5’-AGGAATTCTACATACTTGACAC-

***Analysis of 8-OHdG DNA adducts in colon tissue***

Homogeneous suspension of colon tissue (100 mg) in ~4 ml of genomic DNA buffer was treated with proteinase K (250 µg) at 55 ^°^C for 2 hr. DNA was then extracted with phenol/chloroform/isoamyl alcohol (25:24:1) and precipitated using ethanol. The re-suspended DNA was treated with RNase A (100 µg) and RNase T1 (0.5 µl) for 1 hr at 37 ^°^C to remove traces of RNA, if any. DNA was then digested to nucleosides with nuclease P1 and bacterial alkaline phosphatase and analyzed by HPLC-ECD, as described previously (32). Briefly, HPLC system with two reversed-phase (3.9x150 mm, 5 µm from Delta-Pak 011795, Waters, Milford, MA) columns connected in series and an isocratic pump delivering 10% methanol and 50 mM sodium acetate (pH 5.3) at 0.8 ml/min as the mobile phase was used for separation of 8OHdG adducts. These oxidized DNA adducts were detected with an electrochemical detector consisting of an analytical cell. A UV detector at 290 nm was used to detect dG.

***Measurement of O*** ^6^ ***MeG by HPLC-FLD/DAD***

Isolated DNA (10-30 µg) was subjected to depurination using 60% formic acid at 95 ^°^C for 90 min. The depurinated DNA was then allowed to remain at room temperature and concentrated using a rotary evaporator. The concentrate was redissolved in 200 µl of 0.3 mM formic acid in 10% methanol for further analysis. O^6^MeG and guanine were analyzed by injecting 20-40 µl of sample. The chromatographic system was equipped with a C18 5 µm 100 AU, 250x4.6 mm column, with extended polar selectivity for better resolution. Elution was achieved using a mobile phase consisting of 25 mM ammonium formate in 20% methanol, pH 3.0 pumped at a flow rate of 1 ml/min. Guanine was detected by a diode array detector set at 250 nm, while O^6^MeG was detected using a fluorescence detector set at Ex=285 nm, Em=345 nm. The adduct load was expressed as μmol O^6^MeG/ mol guanine. Calibration curves were prepared for O^6^MeG (0.12-3.03 pmol) and guanine (0.11-2.65 nmol) injected onto the column.

***Electron spin resonance spectroscopy***

In order to understand the nature and intensity of free radical species produced in AOM-administered rat colon mucosa, we have adopted electron spin resonance spectroscopy. The proximal colon segment was collected and the luminal contents were flushed with ice-cold saline. The mucosal scrapings were obtained with a glass slide, frozen in liquid nitrogen, and stored at -80 ^°^C. Electron spin resonance measurements were performed at room temperature using a Varian E-109 spectrometer equipped with a loop gap resonator. Spectra were recorded using 9.1 GHz of microwave frequency, 2 mW of microwave power, 100 kHz of modulation frequency, 2.5 G of modulation amplitude, a field set of 3280 G with a scan range of 100 G, a 4 min scan time, and a time constant of 0.5 sec. Sample solutions contained 25 µl of colon mucosal scrapings, 2 µ1 of the spin trap, and 5,5- dimethyl-l-pyrroline-N-oxide (DMPO), which was added to a final concentration 153 mM. Samples were placed in the cavity of the magnet and spectra recorded 3 min after addition of the DMPO.

***Statistical analysis***

Statistical analyses were undertaken using SPSS version 17.0. The data were expressed as the mean±standard deviation (SD) and analyzed using one-way analysis of variance (ANOVA). A *P*-value<0.05 was considered significant.

**Table 1:** Inhibitory effect of phytate on AOM-induced aberrant crypt foci and tumor incidence in Fisher 344 male rat colon

Treatment	Incidence of ACF (%)	ACF/colon	Crypt multiplicity of ACF				Tumor incidence	
			**1 crypt**	**2 crypts**	**3 crypts**	**≥4 crypts**	**Non-invasive (%)**	**Invasive (%)**
Control	0/8 (0)	—	—	—	—	—	—	—
AOM	20/20(100)	386± 38	125± 14	165± 15	58± 6	38± 4	14(58.3)	10(41.6)
AOM + 1% Phytate	20/20 (100)	218± 19*	87± 8*	92± 9*	32± 4*	7± 2*	3	—
AOM + 2% Phytate	20/20(100)	146± 12**	47± 5**	62± 6**	22± 3**	5± 2*	—	—

NOTE: Data are shown as mean±SD of 20 samples in each group.

*P<0.001, vs AOM (Bonferroni T test). AOM: Azoxymethane, ACF: Aberrant crypt foci

**Figure 2:**
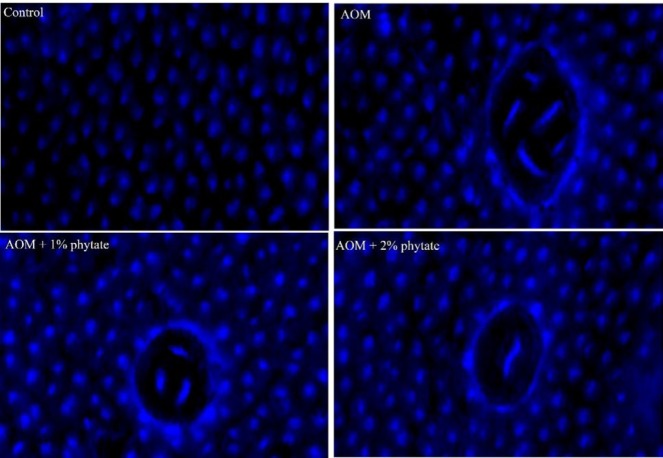
Effect of dietary phytate on AOM-induced ACF in rat’s colon (methylene blue) staining. Normal crypts of control group, ACF consisting of multiple crypts in AOM-administered group. Decrease in number of ACF and crypt multiplicity were observed in AOM-administered rats, but fed 1% and 2% phytate. AOM: Azoxymethane, ACF: Aberrant crypt foci

**Figure 3:**
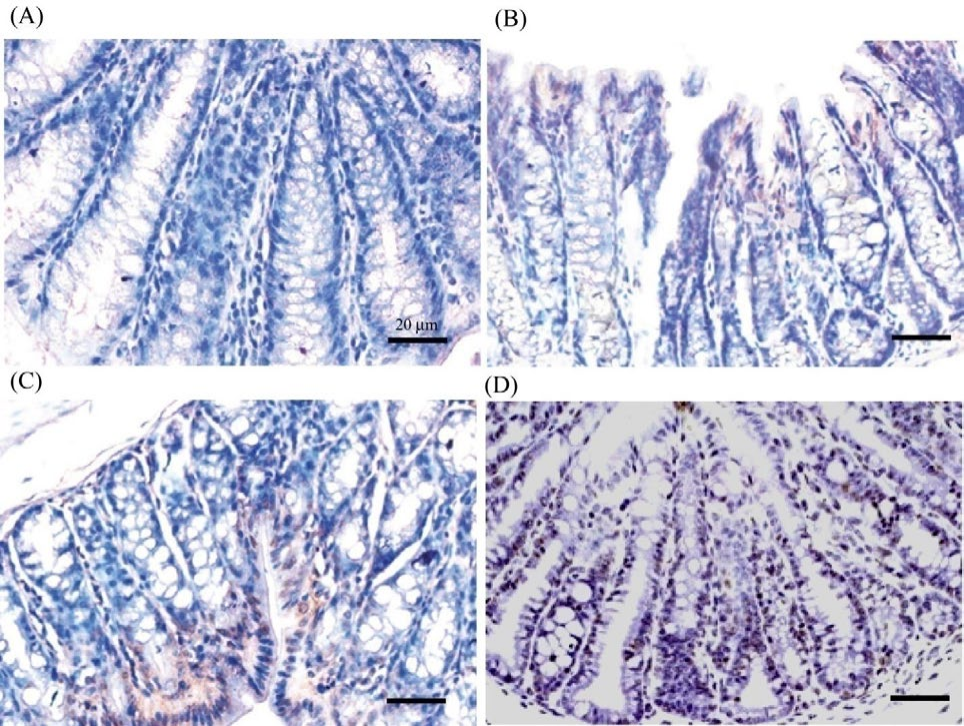
A: Representative images showing TUNEL staining in colon of control (A) and AOM-injected rats (B-D), supplemented with 1% phytate (C) or 2% phytate (D) for 16 weeks post-initiation (×40 objective, scale bar = 20 μm). AOM: Azoxymethane

**Table 2:** Apoptotic index in colonic mucosa of phytate-treated AOM-induced colon cancer

Treatment	Apoptotic cells (%)
Control	3.28 ± 1.04
AOM	2.58 ± 1.12
AOM + 1% phytate	22.50* ± 5.60
AOM + 2% phytate	36.86 **± 6.64

Each value expressed as mean±SD of three determinations. Value in the same column with different superscript letter indicates significant difference by Tukey test (*P<*0.05). Administration of 1% and 2% phytate significantly increased the apoptotic cells compared to the AOM-alone group (*P<*0.05). AOM: Azoxymethane

**Figure 4:**
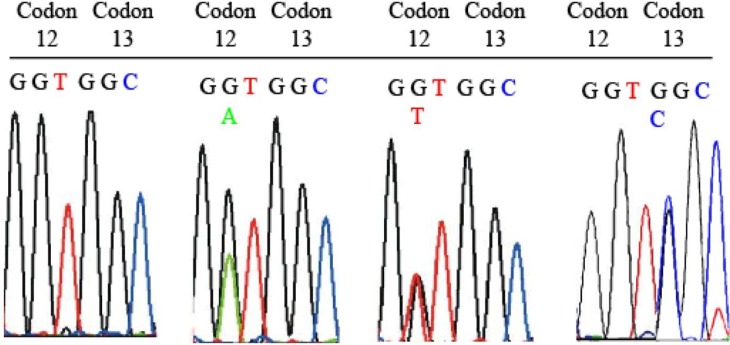
Detection of mutant K-*ras* in DNA from colonic ACF and tumor tissues. Examples of electropherograms indicating point mutations within K-*ras* gene amplified from large intestinal adenomas and carcinomas of F344 rats treated with AOM and fed 1% or 2% dietary phytate. (A) Identification of point mutations in codons 12 and 13 (exons 1 and 2) of the K-*ras* gene. (a) Normal *K-ras* codon 12 (GGT) and codon 13 (GGC); (b) adenoma with mutated codon 13 (GGC>CGC); (c) carcinoma with mutated codon 12 (GGT>GAT). AOM: Azoxymethane

**Table 3:** 8-OHdG and O^6^MedG adducts in the colon mucosa from AOM-administered and phytate-supplemented rats

Treatment	8-OHdG/10^6^ G	O6MeG/10^6^ G
Control	0.15±0.02	-nd-
AOM	0.86*±0.07	0.48*±0.08
AOM+1% Phytate	0.52**±0.05	0.31**±0.06
AOM+2% Phytate	0.38**±0.03	0.18**±0.05

Data are Mean±SD. *P<0.05, vs control; ** *P<*0.05 vs AOM by one-way ANOVA. –nd- is not detectable. AOM: Azoxymethane, O^6^-MeG: O^6^-methyl guanosine, 8-OHdG: 8-hydroxy deoxyguanosine

**Table 4:** K-*ras* mutations in aberrant crypt foci and normal colon mucosa of AOM-treated and phytate-fed rats

Group (n)	Mutation type at Codon 12 (number of ACF/normal mucosa samples analyzed)	Mutation type at Codon 13 (number of ACF/normal mucosa samples analyzed)
Control (8)	GGT-GAT 0% (0/24), GGT-GTT 0% (0/24)	GGC-CGC 0% (0/24)
AOM (20)	GGT-GAT 60% (24/40), GGT-GTT 40% (16/40)	GGC-CGC 18% (7/40)
AOM+1% phytate (20)	GGT-GAT 30% (12/40), GGT-GTT 10% (4/40)	GGC-CGC 0% (0/24)
AOM+2% phytate (20)	GGT-GAT 10% (4/40), GGT-GTT 5% (2/40)	GGC-CGC 0% (0/24)

AOM: Azoxymethane, ACF: Aberrant crypt foci

**Figure 5:**
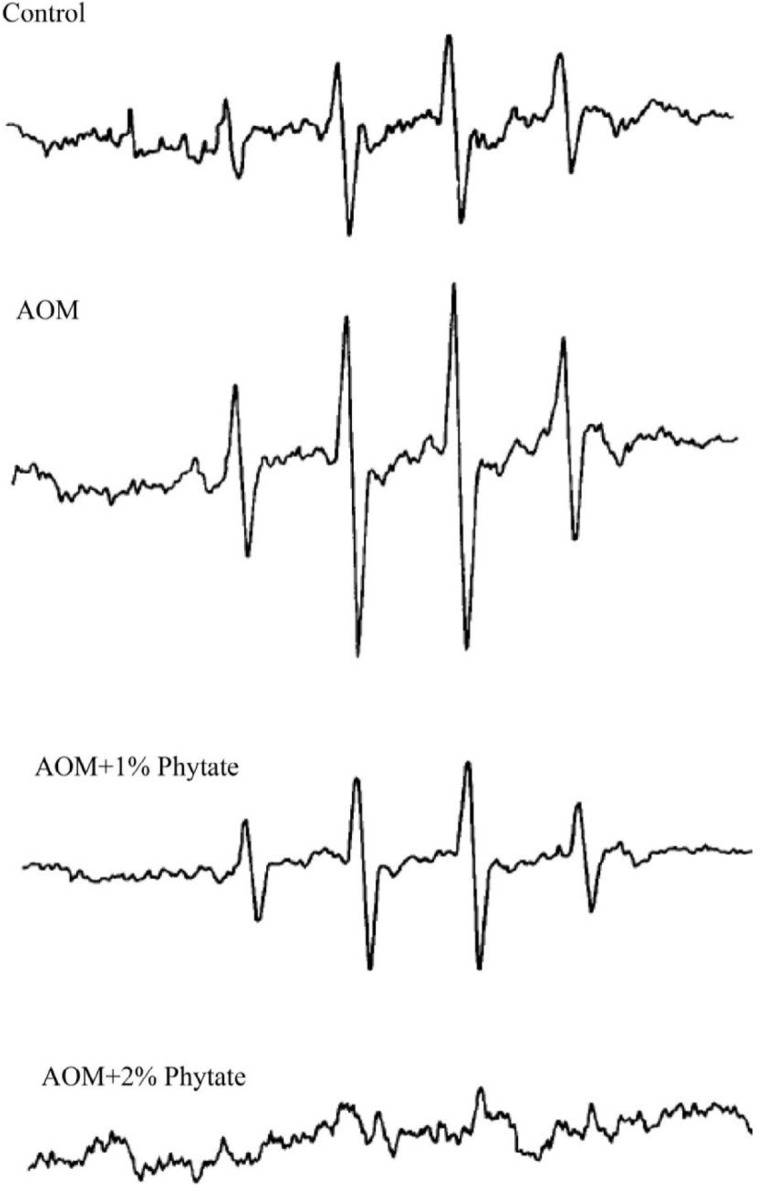
Electron spin resonance spectra of DMPO-OH adduct observed in colon mucosa from AOM-administered rats, with and without different doses of phytate. All the spectra were recorded using 153 mM DMPO and a receiver gain of 2.0 x 105. AOM: Azoxymethane, DMPO: 5,5- dimethyl-l-pyrroline-N-oxide

**Figure 6:**
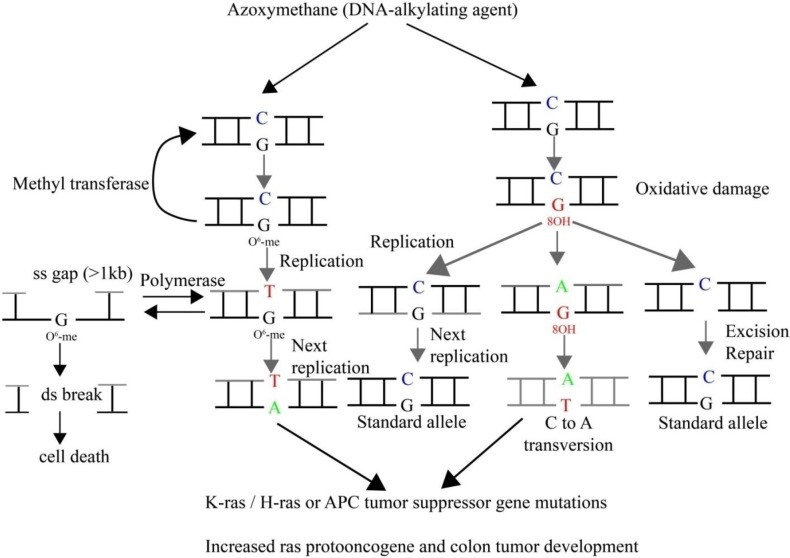
Mechanisms of genotoxicity of azoxymethane. Direct biological effect of methylating agent, azoxymethane, results in O6-meG. The methylated G–T mismatch can form A-T, resulting in G:C to A:T transitions, or may be repaired by methyl transferase. Recombination repair may involve the homologous recombination or non-homologous end joining pathways. Indirect effects through formation of 8-OHdG due to increased oxidative stress may result in C to A transversions or may be repaired by excision. O6-MeG: O6-methyl guanosine, 8-OHdG: 8-hydroxy deoxyguanosine

## Results

***ACF and tumor analysis***

All rats treated with AOM developed ACF, whereas no evidence of ACF could be detected in the colons of vehicle-treated control animals. Normal mucosa showed crypts arranged in parallel with round nuclei and mucin stained blue, whereas hyperplastic ACF showed mucin depletion and elongated nuclei. The data on the modifying efficacy of dietary phytate intervention on further development of colonic ACF and their progression into colonic tumors was studied at 36 weeks, i.e., after 16 weeks of supplementation with different levels of dietary phytate (1 or 2%). The numbers and incidences of ACF along with presence of non-invasive and invasive colon tumors are presented in the Table 1. The average ACF unit over the entire length of colon in the AOM-administered rats was 155±14. The dietary administration of phytate for 16 weeks after initiation with AOM greatly (*P*<0.001) lowered the number of ACF to 87 and 59 with 1% and 2% phytate, respectively. Furthermore, the number of ACF consisting of >4 crypts also decreased significantly (*P*<0.05) in phytate-fed groups as compared to AOM alone–treated group (Figure 2). A total of 8 tumors were observed in the AOM-alone administered animals, which included both invasive (37.5%) and non-invasive (62.5%) tumors. Two animals in AOM-administered and 1% phytate-fed group showed non-invasive tumors. A significant reduction in tumor multiplicity was observed in the group that received 2% phytate. Further, preneoplastic dysplastic ACF number and incidence decreased significantly in both 1% and 2% phytate-fed animals compared to AOM-administered no phytate fed animals.

***Phytate increases apoptotic index in colonic tumors***

In order to determine if phytate supplementation tipped the balance in favor of apoptosis, colon tissue sections were evaluated using TUNEL assay for obtaining apoptotic index. Apoptotix index, as indicated by brown stained nuclei, increased significantly with phytate supplementation as compared to the AOM-alone group (Figure 3). Changes in apoptotic index with phytate supplementation in AOM-administered rats are shown in Table 2. In the AOM-administered rats, the mean number of apoptotic cells in the colon was 2.58% as compared to 3.28% in control rats that received vehicle. Analysis also indicated that the supplementation of phytate led to a progressive increase in apoptotic index in AOM-administered rats (Figure 3). Taken together, supplementation of phytate enhanced the apoptotic rates of colonic tumor cells. 

***Modifying effect of dietary phytate on Ras mutational frequencies in AOM-treated rats***

Table 3 shows the 8-oxodG and O^6^-MeG levels in colon tissues as determined by HPLC in AOM-administered rats and the modifying effect of phytate. The formation of 8-oxodG as well as O^6^-MeG increased significantly in AOM-administered animals compared to control colon tissue. With 1% and 2% phytate supplementation, the DNA adducts significantly decreased in a dose-dependent manner. After AOM exposure, substantially increased quantity of DNA adducts in the colon was observed, whereas consumption of phytate-enriched diets (both 1 and 2%) reduced colonic adduct load compared to no-phytate fed diet.

To characterize and validate the *K-ras* mutations induced by AOM, DNA from paraffin-embedded tissue samples, representing ACFs and tumors was isolated. Automated DNA sequencing results presented in Table 4, from the PCR amplified DNA demonstrated that 60% of the colon tumors from AOM-treated and control diet fed animals showed GGT to GAT transition and 40% of the tumors showed GGT to GTT transversion at codon 12. In addition, 18% of these tumors also showed GGC to CGC transversion at codon 13, as shown in Figure 4. Phytate supplementation at both 1 and 2% lowered the frequency of GGT > GAT to 30 and 10%, respectively. Phytate supplementation also nullified the codon 13 mutations. No mutations were observed at codon 61 in any of the experimental groups. Thus, these results clearly suggest a decrease in *K-ras* mutation frequency with phytate supplementation in AOM-administered animals.

In DMPO-treated AOM-induced colon mucosa, ESR measurements revealed spectra that consisted of a quartet signal (1:2:2:1) with a hyperfine splitting of 14.9 Gauss (splitting constants of a_N_ = a_H_ = 14.9 G), characteristic of a DMPO adduct produced by spin trapping of a hydroxyl radical. A similar spectrum was seen in control colon mucosa but was of far less intensity compared to AOM-administered colon mucosa. The signal intensity decreased significantly in the colon mucosa from AOM-administered animals treated with 1% phytate, whereas 2% phytate treatment led to complete inhibition of the signal. DMPO-OH adduct formation observed in AOM-administered colon mucosa was found to be reduced with 1% phytate and eliminated with 2% phytate. 153 mM DMPO was used. Representative spectra from each group recorded are shown in Figure 5.

## Discussion

Numerous anecdotal reports indicated that phytate has anticancer properties. Antineoplastic activity of phytate has been attributed to altered gene function (34), cell cycle inhibition (35), modulation of cellular signal transduction (36, 37), antioxidant function through chelation of iron at the cellular level (38, 39). In this study, we investigated the role of dietary phytate in tumor development and apoptosis via accumulation of 8-OHdG, O^6^-MeG adducts and free radical generation. The experimental design employed continued dietary phytate supplementation for 16 weeks, after formation of ACF and therefore the mechanisms independent of detoxification effect of the carcinogen were the focus of the study. Metabolic products of AOM capable of methylating the DNA result in the development of dysplastic aberrant crypts and their progression to adenomas, followed by adenocarcinoma development. In this experiment, all the AOM-administered rats developed ACF at the end of 20 weeks, thus establishing the carcinogenic potential of AOM, despite dietary treatment with phytate being in progress. Further, the effect of dietary phytate on tumors was studied as a measure of growth inhibitory potential during the tumor development phase, as it is known that aberrant crypts with highly multiplicity show increased tumor incidence (40). Dietary phytate significantly lowered the number of aberrant crypts and their multiplicity, thereby ameliorating the tumor incidence. A significant decrease in tumor incidence and multiplicity, with both concentrations of 1% and 2% phytate was observed, which was consistent with previously reported chemopreventive efficacy of dietary phytate (1, 2). 

AOM administration is known to increase the ROS due to a decrease in antioxidant enzyme activity and depletion of reduced glutathione (GSH) (41, 42). AOM was also shown to increase cellular dichlorofluorescein (DCF) oxidation and malondialdehyde levels, indicating colonic cellular oxidative damage in association with inflammatory effect. These changes in cellular oxidative damage were ameliorated to a great extent by natural extracts (43). Given the importance of ROS in triggering uncontrolled proliferation (44), and the capacity of phytate to exert both antioxidant, anti-inflammatory effect (45) (39), we have studied if dietary phytate exerts anti-mutagenic effects in lowering the tumor burden. Colon epithelial cells lining the bowel divide rapidly and exhibit enhanced metabolic rate, making them more susceptible to increased oxidative burden (46). Colonocytes residing in the lower crypt zone with rapid proliferation rates were found to be more sensitive to hydrogen peroxide when compared to differentiated cells on the surface of the crypt (47). Thus, the stem cell progenitors and dividing daughter cells appear to be more sensitive to the redox changes in the gut mucosa as well as in the lumen. Moreover, the DNA in the proliferating cells is present as a single strand in the S-phase of the cell cycle, giving it exceptional sensitivity towards damaging agents and resulting in mutations, which are irreparable (47). The DNA damage can result in cell cycle arrest or transcription, replication errors, and genomic instability, all of which are involved in colon carcinogenesis (48). 

Mutations in the *K-ras* oncogene were suggested to be present in large adenomas, but not in small adenomas (49). ACF that are considered putative preneoplastic lesions were also shown to accumulate mutations in *K-ras* (50-54). Thus, following the mutational frequency of *K-ras* enables insights into its role in carcinogenesis as well as to observe the modifying effect of the intervention. Pooled ACF from the AOM-administered rats showed *K-ras* codon 12 mutations (G to A and G to T) in 25% of rats after 12 weeks of induction with AOM. The frequency increased with the duration of carcinogen exposure. 37.5% and 70% of the rats showed *K-ras* mutations at 20 and 32 weeks, respectively (19, 55). *K-ras* had almost 40-60% frequency of mutations at codon 12 in colon tumors from AOM-administered rats in the present study. G to A transitions and G to T transversions could not be observed at codon 12 in the control mucosa. On the other hand, AOM-treated rats exhibited not only G to A transition as previously reported (11, 56-58), but also G to T transversion. This is one of the novel findings of the present study. In addition, this study also observed a significantly greater rate of K-*ras* mutation rate compared to the previous reports (56-58). AOM triggers K-*ras* gene mutation from G to A at codon 12 due to O^6^-MeG adducts (59). This transition changes Gly to Asp, resulting in activation of the K-ras protein, which can potentially activate the downstream signaling pathways, namely mitogen activated protein kinase (MAPK) and phosphoinositide 3-kinase (PI3K)/Akt. Based on the previous reports and the present study, it is likely that the K-*ras* mutations spectrum is largely affected by the specific actions of the carcinogen in a tissue, strain and species specific manner. Various other factors, including the site of accumulation of adducts, selection frequency, repair efficiency of mismatched bases do govern the mutational spectrum. For example, AOM in rats was reported to induce G to A mutation exclusively, while sporadic human colon tumors exhibit not only G to A, but also G to T and G to C mutations (11). Similarly, AOM-treated rats also showed G to C mutation at codon 13 (GGC to CGC), leading to substitution of Gly by Arg. Genotoxicity and mutagenicity mechanism of AOM is shown schematically in Figure 6. 

Because phytate is considered to lower bioavailability of iron, as a potential pro-oxidant, we hypothesized that phytate may be inhibiting colon carcinogenesis in this model by affecting specific gene mutations associated with ROS production. ROS are known to cause oxidative modifications, leading to carcinogenesis due to the mispair/mutagenic potential of the modified base. To critically understand if dietary phytate acts as an antioxidant under the experimental conditions *in vivo*, we have undertaken spin trapping studies with DMPO and characterized the type of radicals formed. Our present study clearly demonstrated that dietary phytic acid (2%) clearly inhibited the production of hydroxyl radicals. This study as well as the previous studies suggest that phosphate moieties of IP6 (phytate) chelate iron and thus reduce the radical formation (60). Control animals receiving adequate dietary iron also showed the quartet signal, indicating that basal levels of ROS are indeed required for controlled proliferation. However, the enhanced production of ROS in AOM-administered animals receiving the same level of dietary iron indicates interactions between the carcinogen (AOM) and the iron to produce more ROS. Similarly, dietary phytate supplementation in the AOM-administered animals further suggests interactions between iron and phytate, towards reducing the signal intensity. The reduction and elimination of signal intensity probably relate to the phytate/iron molar ratios.

Increased ROS with AOM administration as evidenced by ESR corresponds to increased frequency of transversion mutations in *K-ras*. Damage of DNA by ROS forms oxidative DNA adducts, (61, 62). 8-hydroxydeoxyguanosine (8-OHdG) is capable of pairing up with adenine and results in a GC:TA transversion mutation (63). In addition to ROS, AOM is known to cause O^6^-methyl guanine adduct formation, which if unrepaired by methylguanine methyl transferase (MGMT) also results in G to A transitions and ultimately K-*ras* mutations (19, 55). Alkylated metabolites of AOM, namely methyldiazonium ion or methyldiazohydroxide, is thought to be responsible for inducing mutations at the second nucleotide position of codon 12. Methylation of guanine at the O^6^ position within the protooncogene domain (CCTGG) and mispairing with thymidine during DNA replication appears to be the underlying process. The base change may not be efficiently repaired by the DNA polymerase, either due to an effect of adjoining bases on the miscoding or inefficient repair by MGMT (64, 65). Persistent increase in mutation rates makes the cells unstable while allowing them to accumulate aberrations that afford proliferative advantage. Increased accumulation of 8-OHdG and O^6^-MeG adducts observed in AOM-induced colon mucosa also points to the induction of DNA damage. These changes are inherent to various cancers and although appear early but persist during colon carcinogenesis (66, 67). A significant reduction of both 8-OHdG and O^6^-MeG adduct load in the rat colon with consumption of dietary phytate in AOM-administered animals indicates either their removal by specific enzymatic mechanisms or eradication through cellular apoptosis. Increased apoptotic index, as shown by TUNEL staining, with phytate supplementation supports the latter. 

Mutations evoked by AOM can induce genomic instability, and hence increase the sensitivity to luminal soluble iron, which can act as a tumor promoter. Iron may contribute to the selection of pre-existing mutation evoked by AOM or result in additional mutations, through generation of hydroxyl radicals via Fenton’s reaction in the multistep carcinogenesis. Dietary phytate that can insolubilize iron in the diet, although increases unabsorbed iron in the lumen of gastrointestinal tract appears to reduce the progression to tumor, which is associated with lowered oxidative stress and reduced mutation frequency.

## Conclusion

These studies clearly suggest that dietary phytate protects against AOM-induced pro-mutagenic adduct accumulation in the colon. Increased apoptotic rates observed with dietary phytate appears to remove DNA-damaged cells. Phytate decreased the incidence of adenocarcinomas with *K-ras* codon 12 mutations in a dose-dependent manner. Reduction in *K-ras* mutational frequency with increasing dosage of dietary phytate was found to be statistically significant. To our knowledge, this is the first report, wherein phytate has been shown to decrease the incidence of *K-ras* mutations in AOM-administered rats.
